# Successful demonstration of simultaneous PET/MR Imaging with a RF-penetrable PET insert

**DOI:** 10.1186/2197-7364-2-S1-A17

**Published:** 2015-05-18

**Authors:** Brian Lee, Alexander Grant, Chen-Ming Chang, Gary Glover, Craig Levin

**Affiliations:** Stanford University, Palo Alto, CA USA

The integration of PET’s ability to visualize and quantify molecular signatures and MRI’s excellent anatomical information shows great promise to be a powerful tool for disease characterization. However, the high cost of simultaneous PET/MRI has limited its availability. To address this problem, we have developed an RF-penetrable PET insert that can be placed in the bore of any MRI system without requiring modifications to the latter. The PET insert consists of 16 PET detector modules within Faraday cages in a 32 cm inner diameter ring pattern with small gaps. These gaps, along with electro-optical signal transmission technology that allows the PET insert to electrically float relative to the MRI RF ground, enable RF fields to pass through the PET ring with some attenuation. In this study, we investigated the feasibility of the RF-penetrable PET insert placed inside a 3T MR system using the body coils for the RF transmit/receive coil. We analyzed the transmit and receive penetration of the RF fields by acquiring B1 maps and MR images. When the PET insert was placed inside the MRI bore, compared to the case with no PET insert, the mean B1 field amplitude (transmitted RF field) was attenuated by -3.47 dB. The SNR (received MR signal) was attenuated by similar factors of -3.4 dB and -3.9 dB with GRE and FSE sequences, respectively. In addition, we acquired simultaneous PET/MR images. The PET images acquired inside the MRI bore were equivalent to those acquired outside the MR system. We have shown that the RF-penetrable PET technology allows the RF field of an MR body coil to penetrate into the field-of-view, enabling acquisition of simultaneous PET/MR images via a PET insert, without modification to the MR system.

